# Amine-responsive Rhodopsin-like GPCRs in ocular pathology: mechanistic associations, candidate biomarkers, and therapeutic potential

**DOI:** 10.3389/fphys.2026.1923694

**Published:** 2026-09-22

**Authors:** Han Yu, Jia Li, Jike Song, Hongsheng Bi

**Affiliations:** 1 Shandong University of Traditional Chinese Medicine, Jinan, China; 2 Shandong Provincial Key Laboratory of Integrated Traditional Chinese and Western Medicine for Prevention and Therapy of Ocular Diseases; Shandong Academy of Eye Disease Prevention and Therapy, Jinan, China; 3 Affiliated Eye Hospital of Shandong University of Traditional Chinese Medicine, Jinan, China

**Keywords:** adrenoceptors, dopamine receptor, GPCRs, muscarinic acetylcholine receptors, myopia, ocular pathologies, regulatory mechanisms

## Abstract

Rhodopsin-like G protein-coupled receptors (GPCRs) play a pivotal role in ophthalmic pathologies, and recent studies have substantially advanced our understanding of their underlying molecular mechanisms. Amine-responsive Rhodopsin-like GPCRs (ARh-GPCRs), encompassing adrenoceptors, dopamine receptors, muscarinic acetylcholine receptors, histamine receptors, serotonin receptors, melatonin receptors, and trace amine-associated receptors, are involved in a variety of ocular pathological processes and have emerged as key therapeutic targets. Elucidating their functional roles, alongside those of their ligands and downstream signaling macromolecules, holds considerable potential for the development of candidate biomarkers for early diagnosis, disease classification, prognostication, and predicting treatment response in specific ocular disorders. Despite significant progress, a comprehensive integration of their dual candidate biomarker-therapeutic roles across diverse ocular disorders remains largely unexplored. This narrative review summarizes and critically integrates current evidence on the regulatory roles and therapeutic potential of these receptors in ocular pathologies, with particular attention to their mechanistic involvement, candidate biomarker potential, and therapeutic relevance. Furthermore, we summarize the current research limitations and propose future directions to enhance the understanding of their molecular mechanisms, ultimately facilitating the development of precise diagnostic and therapeutic strategies.

## 1 Introduction

G Protein-Coupled Receptors (GPCRs) are functionally diverse transmembrane proteins that form the largest family of cell membrane receptors in the human genome (Fredriksson et al., 2003; Gacasan et al., 2017). They regulate numerous physiological processes, including vision, neural conduction, development, and immune responses. GPCRs mediate approximately two-thirds of endogenous physiological responses (Foster et al., 2019) and serve as targets for one-third of clinical drugs (Addis et al., 2024; Lorente et al., 2025), representing the most important category of drug targets currently marketed or in development (Müller et al., 2012). The Rhodopsin-like GPCR family, also known as Class A GPCRs, is named after rhodopsin, a prototypical receptor in which retinal is covalently bound to opsin through a Schiff base linkage (Chen et al., 2022). These receptors transduce extracellular signals via a 7-transmembrane α-helical structure (TM1-TM7), activating second messengers and downstream pathways (Lagerström and Schiöth, 2008). This class recognizes a diverse range of ligands, from small molecules (e.g., protons, neurotransmitters) to large molecules (e.g., peptides, glycoproteins).

Amine-responsive Rhodopsin-like GPCRs (ARh-GPCRs) are defined as Rhodopsin-like receptors that use biogenic amines (e.g., acetylcholine, 5-hydroxytryptamine, and trace amines) as endogenous ligands (**Table 1**). These receptors recognize ligands through highly conserved orthosteric binding pockets and share substantial mechanistic similarities. The fully conserved D3.32 residue in TM3 (>99% conserved in human Class A GPCRs) forms a critical salt bridge with the ligand’s positively charged amino group. Simultaneously, Y7.43 and W7.40 in TM7 synergistically stabilize this interaction through hydrogen bonding and π-π stacking. This shared structural framework explains the prevalence of polypharmacology, where single drug molecules interact with multiple amine receptors due to similar binding modes. Consequently, it provides a key structural basis for multi-target drug design (Kooistra et al., 2013). Given their broad expression in ocular tissues and central roles in neurotransmission, vascular regulation, immune responses, and cellular homeostasis, ARh-GPCRs are increasingly recognized as important regulators of ocular physiology and disease (Chen and Palczewski, 2016).

**Table 1:** Functional architecture of ARh-GPCRs.

Receptors	Ligands	Subtypes	Gene	Distributions	Species	Disease context/model	Core functionalities	Human clinical evidence
Adrenoceptors	Epinephrine, Norepinephrine	α_1_A-AR	ADRA1A	Iris smooth muscle, Ciliary body vascular smooth muscle, Retina, Corneal epithelium, Conjunctiva	Rat, Mouse	Retinal vascular physiology, Retinal detachment, Pupillary regulation, Allergic conjunctivitis, Corneal epithelial homeostasis, Autoimmune retinal inflammation	Vascular tone regulation (Mori et al., 2011), Retinal structure and function protection (Li et al., 2019), Mydriasis (Raczak-Gutknecht et al., 2017), Inflammation (Liu et al., 2020), Corneal epithelial regeneration (Musayeva et al., 2018), Blood-retinal barrier homeostasis (Stofkova et al., 2019), Angiogenesis, Pain, Intraocular pressure regulation	No
		α_1_B-AR	ADRA1B	Ciliary body vascular endothelium, Choroidal capillaries, Retina	Mouse	Retinal vascular dysfunction	Blood-retinal barrier homeostasis (Böhmer et al., 2014), Inflammatory vascular leakage	No
		α_1_D-AR	ADRA1D	Corneal marginal endothelial cells, Scleral vascular smooth muscle, Retinal pigment epithelium	Rat, Human cell line (ARPE-19)	Retinal vascular regulation, Diabetic retinopathy	Vascular tone regulation (Mori et al., 2011), Retinal structure and function protection (Giuffrida et al., 2025), Angiogenesis, Pain	Yes
		α_2_A-AR	ADRA2A	Nonpigmented ciliary epithelium, Retina, Optic nerve	Rat, Chick, Human cell line (MIO-M1), Rabbit	Diabetic retinopathy, Retinal detachment, Myopia, Retinal injury-related glial response, Aqueous flare and IOP elevation	Retinal oxidative stress (Jung et al., 2024; Li et al., 2019), Refractive error (Carr et al., 2019), Intraocular pressure regulation (Harun-Or-Rashid and Hallböök, 2019; Nakamura-Shibasaki et al., 2016), Aqueous humor production regulation, Optic neuroprotection	Yes
		α_2_B-AR	ADRA2B	Iris, Corneal epithelium	Mouse	Dry eye disease	Ocular surface immunoregulation (Fan et al., 2025)	No
		α_2_C-AR	ADRA2C	Trabecular meshwork, Corneal epithelium	Mouse	Dry eye disease	Ocular surface immunoregulation (Fan et al., 2025)	No
		β_1_-AR	ADRB1	Inner nuclear layer, Retinal vascular endothelium, Optic nerve	Mouse	Proliferative retinopathy	Angiogenesis (Dal Monte et al., 2014), Retinal fibrosis, Optic disc edema	No
		β_2_-AR	ADRB2	Trabecular meshwork, Corneal epithelium, Ciliary muscle, Corneal epithelium, Choroid, Retina, Meibomian gland	Mouse, Human cell line (Retinal endothelial cells, HCECs, HMGECs, Müller cells, RPE cells and neutrophils), Human	Diabetic retinopathy, Dry eye disease, Choroidal neovascularization, Retinopathy of prematurity, Proliferative retinopathy, Glaucoma, Ocular surface damage, Meibomian gland dysfunction	Inflammation (Qu et al., 2025; Shi et al., 2017), Angiogenesis (Cammalleri et al., 2024; Dal Monte et al., 2014; Droho et al., 2019; Lavine et al., 2017; Makhoul et al., 2013; Ristori et al., 2011), Intraocular pressure regulation (Hohberger et al., 2021a; Hohberger et al., 2021b), Wound healing (Yuan et al., 2021), Meibomian gland lipid synthesis (Jun et al., 2022), Tear secretion (Zhang et al., 2023), Aqueous humor outflow regulation	Yes
		β_3_-AR	ADRB3	Corneal marginal endothelial cells, Scleral vascular smooth muscle, Retinal pigment epithelium, Iris	Human cell line (hRECs and MIO-M1), Mouse	Hypoxia-induced retinal neovascularization	Hypoxia and Angiogenesis (Amato et al., 2022; Cammalleri et al., 2023; Lucchesi et al., 2025), Stem cell proliferation, Iris Cyst	Yes
Dopamine Receptors	Dopamine	D_1_R	DRD1	Retinal amacrine cell, Retinal ganglion cell, Bipolar nerve cell, Retinal pigment epithelium	Mouse, Rat, Human primary scleral fibroblasts, Guinea pig	Diabetic retinopathy, Retinal light adaptation, Glaucoma, Myopia	Visual signal modulation (Flood et al., 2018; MacIsaac et al., 2024; Mazade et al., 2019), Regulation of RGC excitability (Cui et al., 2017; Flood and Eggers, 2021; Li et al., 2016; Yin et al., 2023), Refractive error(S. Chen et al., 2017; Guan et al., 2025; Jiang et al., 2014; Shu et al., 2023; Zhang et al., 2018), Ocular growth and development	Yes
		D_2_R	DRD2	Retinal pigment epithelium, Retinal ganglion cell, Retinal amacrine cell, Nonpigmented ciliary epithelium	Rat, Tree shrew, Human primary scleral fibroblasts, Mouse, Guinea pig	Retinal visual signal processing, Myopia, Retinitis pigmentosa, RPE–choroid circadian rhythm regulation	Regulation of RGC excitability (Jensen, 2015; Yin et al., 2020, 2023), Refractive error (Guan et al., 2025; Huang et al., 2022; Ward et al., 2017; Wei et al., 2023a; Wei et al., 2023b), Retinopathic fibrosis (Jensen, 2015), Ocular growth and development (Syambani Ulhaq, 2020), Circadian rhythms (Baba et al., 2017), Aqueous humor outflow regulation, Inflammation	Yes
		D_3_R	DRD3	Ciliary body	Mouse, Rat	Glaucoma	Regulation of intraocular pressure (Bucolo et al., 2012; Chu et al., 2000)	No
		D_4_R	DRD4	Retinal amacrine cell, Retinal ganglion cell, Photoreceptors	Mouse, Guinea pig	Diabetic retinopathy, Myopia	Visual signal modulation (MacIsaac et al., 2024), Refractive error (Guan et al., 2025; Ward et al., 2017)	No
		D_5_R	DRD5	Retinal ganglion cell, Retinal pigment epithelium	Human cell line (ARPE-19)	Retinal fibrosis	RPE fibrotic remodeling (Gao et al., 2022)	Yes
Muscarinic Acetylcholine Receptors	Acetylcholine	M_1_R	CHRM1	Retinal amacrine cell, Retinal ganglion cell, Sclera	Mouse, Rat, Tree shrew, Guinea pig, Rabbit	Glaucoma, Myopia	Regulation of RGC excitability (Laspas et al., 2019; Tan et al., 2014), Refractive error (Arumugam and McBrien, 2012; Tao et al., 2018), Oxidative stress and apoptosis (Yu et al., 2021), Visual signal modulation	No
		M_2_R	CHRM2	Ciliary muscle, Retina, Scleral collagen	Mouse, Guinea pig	Myopia	Refractive error (Barathi et al., 2013; Zhou et al., 2025), Ciliary muscle relaxation, Ocular growth and development	No
		M3R	CHRM3	Iris sphincter, Ciliary muscle, Corneal epithelium, Lacrimal gland, Arteria ophthalmica	Human, Syrian hamster, Rabbit, Rat, Mouse	Myopia, Glaucoma, Dry eye disease, Ophthalmic artery vascular regulation	Refractive error (Lin et al., 2012), Intraocular pressure regulation (Yu et al., 2021), Tear secretion (Musayeva et al., 2021), Vascular tone regulation (Gericke et al., 2014), Oxidative stress	Yes
		M_4_R	CHRM4	Retinal ganglion cell	Mouse	Myopia	Ocular growth and development (Laspas et al., 2015)	No
		M_5_R	CHRM5	Retina, Choroid, Sclera, Iris	Tree shrew, Fish	Myopia	Ocular growth regulation (McBrien et al., 2009; Phatarpekar et al., 2005)	No
Histamine Receptors	Histamine	H_1_R	HRH1	Corneal epithelium, Conjunctival epithelial cells, Ciliary muscle, Retinal vascular endothelium	Human, Mouse	Graves’ ophthalmopathy, Glaucoma	Inflammation (Virakul et al., 2016), Retinal neuroprotection (Chen et al., 2022), Angiogenesis and vascular permeability, Intraocular pressure regulation	Yes
		H_2_R	HRH2	Retinal vascular endothelium, Choroidal vasculature, Ciliary muscle	Rabbit	Ocular vascular regulation	Vascular regulation (Gastinger et al., 2006)	Yes
		H_3_R	HRH3	Retinal amacrine cell, Retinal ganglion cell, Optic nerve	Rabbit	Glaucoma	Aqueous humor outflow regulation (Sgambellone et al., 2024), RGC neuroprotection (Lanzi et al., 2019), Visual signal modulation, Intraocular pressure regulation	No
		H_4_R	HRH4	Ocular surface, Cornea, Ciliary muscle, Retinal pigment epithelium, Retinal neurons, Retinal vascular endothelium	Human, Human cell line (patient serum/vitreous samples, ARPE-19), Mouse	Diabetic retinopathy, Macrophage immune regulation	Angiogenesis and vascular permeability (Czerner et al., 2014; Kwon et al., 2024; Lee et al., 2020), Inflammation	Yes
Serotonin Receptors	Serotonin	5-HT_1_AR	HTR1A	Ciliary muscle, Iris smooth muscle, Inner nuclear layer, Cornea	Zebrafish, Rat	Optic nerve injury, Glaucoma	Retinal neuroprotection (Saied-Santiago et al., 2025; Zhou et al., 2019a; Zhou et al., 2019b), Regulation of visual development (Masson, 2019), Aqueous humor regulation, Intraocular pressure regulation	Yes
		5-HT_1_BR	HTR1B	Inner retina, Retinal ganglion cells	Mouse	Retinal functional regulation	Regulation of RGC excitability (Gibson et al., 2025)	No
		5-HT_1_DR	HTR1D	Retina	Mouse	Retinal signaling	Potential modulation of retinal serotonergic signaling (Gibson et al., 2025)	No
		5-HT_2_AR	HTR2A	Corneal epithelium, Conjunctival epithelial cells, Iris smooth muscle, Ciliary muscle, Retinal vascular smooth muscle	Guinea pig, Mouse, Human cell line(limbal cells)	Myopia, Visual loss, Aniridia	Regulation of visual development (Li et al., 2016; Lombaert et al., 2018), Corneal developmental homeostasis (Oved et al., 2021), Aqueous humor secretion, Pupillary regulation, Vascular tone regulation	Yes
		5-HT_2_BR	HTR2B	Corneal epithelium, Nonpigmented ciliary epithelium, Retinal vascular endothelium	Human cell lines (uveal melanoma), Human, Mouse	Uveal melanoma, Retinogeniculate pathway development	Tumor metastasis (Benhassine et al., 2022; Benhassine and Guérin, 2018; Le-Bel et al., 2019; Weidmann et al., 2018), Regulation of visual development (Kolodziejczak et al., 2015), Corneal developmental homeostasis, Aqueous humor production regulation, Retinal vascular development	Yes
		5-HT_2_CR	HTR2C	Retinal amacrine cell, Retinal ganglion cell, Nonpigmented ciliary epithelium	Mouse	Retinal ganglion cell development and function	Regulation of visual development (Trakhtenberg et al., 2017), Visual signal modulation	No
		5-HT_4_R	HTR4	Photoreceptors, Nonpigmented ciliary epithelium, Retina	Mouse	Light-induced photoreceptor degeneration	Modulation of photoreceptor survival(Y. Chen et al., 2013)	No
		5-HT_5_R	HTR5A	Limited ocular evidence				
		5-HT_6_R	HTR6	Limited ocular evidence				
		5-HT_7_R	HTR7	Limited ocular evidence				
Melatonin Receptors	Melatonin	MT_1_R	MTNR1A	Photoreceptors, Nonpigmented ciliary epithelium	Human, Xenopus tropicalis, Mouse, Rat, Rabbit	Uveal melanoma, Rod photoreceptor degeneration, Age-related photoreceptor degeneration, Uveitis, Retinal circadian homeostasis, Glaucoma	Tumor metastasis (Hagström et al., 2024), Melanopsin-mediated circadian photoentrainment (Gianesini et al., 2016; Wiechmann et al., 2020), Inflammation (Yuksel et al., 2023), Retinal structure and function protection (Laurent et al., 2017), Intraocular pressure regulation (Alkozi et al., 2020; Martínez-Águila et al., 2020; Spadoni et al., 2018), Visual signal modulation, Aqueous humor regulation	Yes
		MT_2_R	MTNR2A	Inner nuclear layer, Corneal epithelium, Choroidal blood vessel	Mouse, Rabbit, Human, Cell systems	Glaucoma, Ocular surface inflammation	Intraocular pressure regulation (Alkozi et al., 2020; Martínez-Águila et al., 2020; Spadoni et al., 2018), Corneal epithelial wound healing (Jin et al., 2022; Yoon et al., 2024), Visual signal modulation, Oxidative stress, Blood flow regulation	Yes
Trace Amine-Associated Receptors	Trace Amine	TAAR1	TAAR1	Corneal epithelium, Inner nuclear layer, Iris-Ciliary body, Optic nerve	Human, Rat, Rabbit	Primary glaucoma	Intraocular pressure regulation (Gowtham et al., 2023), Wound healing	Yes
		TAAR5	TAAR5	Limited ocular evidence				
		TAAR6	TAAR6	Limited ocular evidence				
		TAAR8	TAAR8	Retinal pigment epithelium	RPE cell	RPE cellular homeostasis	Regulation of RPE migrasome formation (Kim et al., 2024)	No
		TAAR9	TAAR9	Limited ocular evidence				

Dysregulation of ARh-GPCR signaling has been implicated in multiple ocular pathological processes, including pathological axial elongation, retinal neovascularization, retinal ganglion cell apoptosis, and uveal melanoma progression. Conversely, their agonists and inhibitors counteract pathology in corresponding diseases. These mechanisms underscore these receptors’ pivotal role in ophthalmic disease pathogenesis and their central position as druggable targets for precision interventions. Eye diseases and related mechanisms involving these different receptor subtypes were summarized and presented in **Figure 1**.

**Figure 1:**
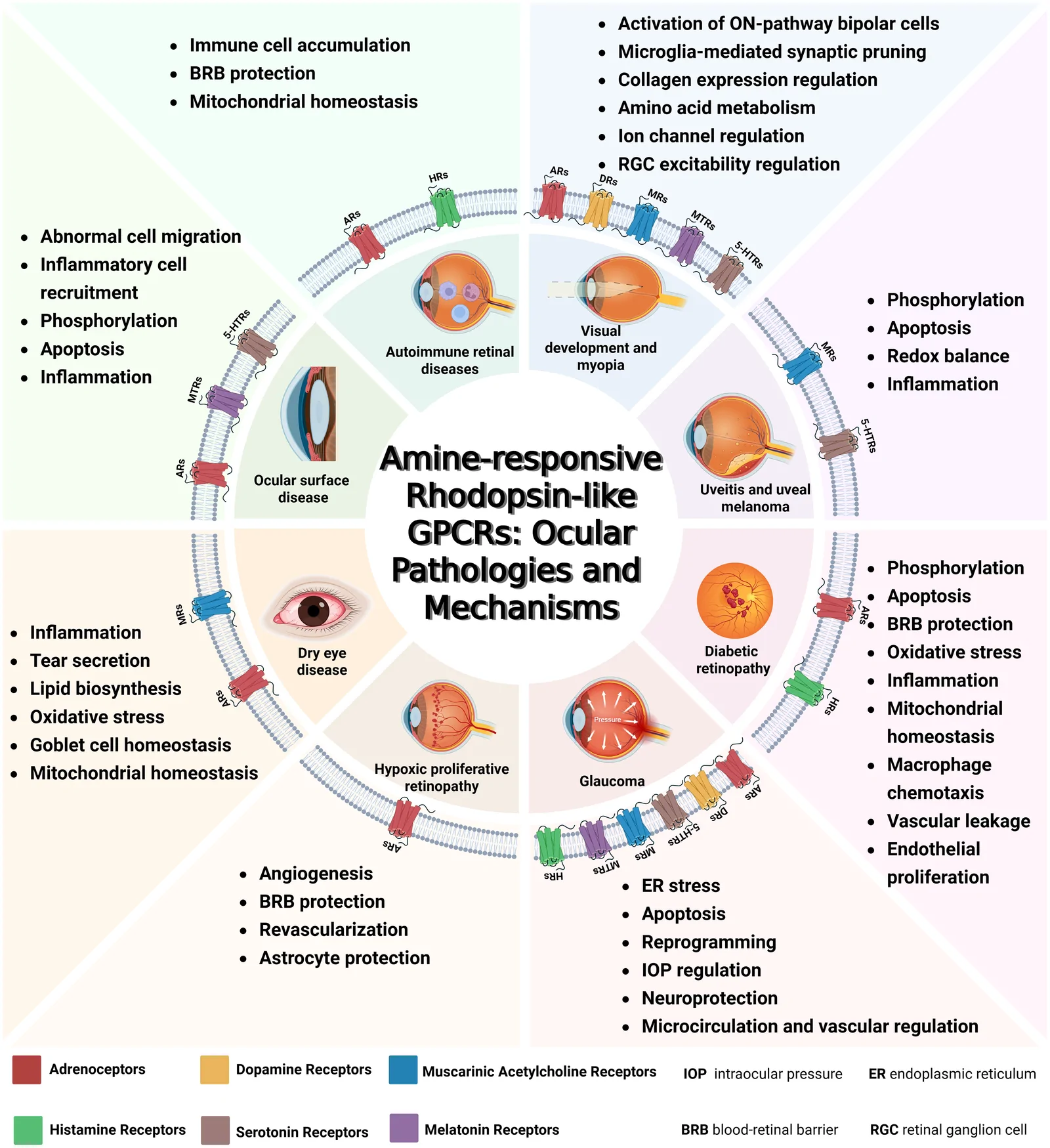
Eye diseases and related mechanisms involving different ARh-GPCRs.

Despite significant advances in mechanistic research in recent years, the dual function of Amine-responsive Rhodopsin-like GPCRs as both diagnostic candidate biomarkers and therapeutic targets across various ocular diseases remains under-evaluated and has not yet been comprehensively integrated. Consequently, this review focuses on key members of the ARh-GPCR family, including adrenergic receptors (ARs), dopaminergic receptors (DRs), muscarinic acetylcholine receptors (mAChRs), histamine receptors (HRs), serotonin receptors (5-HTRs), melatonin receptors (MTNRs), and tryptamine-associated receptors (TAARs). It summarizes the foundational research of the past decade while prioritizing the integration of significant experimental and translational advances from the last five years. For the first time, we adopt a cross-disease, cross-receptor-family systems perspective to evaluate these receptors’ dual role as ‘diagnostic candidate biomarkers and therapeutic targets’ in ophthalmic diseases, rigorously analyzing the key physiological and pathological pathways they mediate alongside associated therapeutic strategies. Building upon this foundation, the paper proposes an integrated research framework centered on multi-target and network regulation. It simultaneously summarizes the primary limitations of current research and outlines future research directions poised to address existing knowledge gaps.

## 2 Adrenoceptors as mechanistic hubs across many ocular diseases

Adrenoceptors (ARs), prototypical ARh-GPCRs, bind catecholamine neurotransmitters norepinephrine (NE) and epinephrine (Epi). They are classified as α_1_-AR (α_1_A, α_1_B, α_1_D), α_2_-AR (α_2_A, α_2_B, α_2_C), and β-AR (β_1_, β_2_, β_3_) (Wu et al., 2021). α_1_-AR couples to Gαq/11; α_2_-AR to Gαi/o; and β-AR to Gαs. In the eye, ARs localize primarily to the ciliary body, trabecular meshwork, and retina, where they regulate aqueous humor dynamics, neuroprotection, and ocular surface homeostasis (Ruan et al., 2024). The physiological processes and molecular mechanisms of eye diseases affected by adrenoceptors were summarized and presented in **Figure 2**.α-AR mainly regulate myopia, neuroprotection and immune inflammation; while β-AR play a dominant role in hypoxic lesions through the HIF/VEGF pathway. These two types of receptors jointly regulate the homeostasis of the ocular surface and the progression of glaucoma, forming a multi-target therapeutic network.

**Figure 2:**
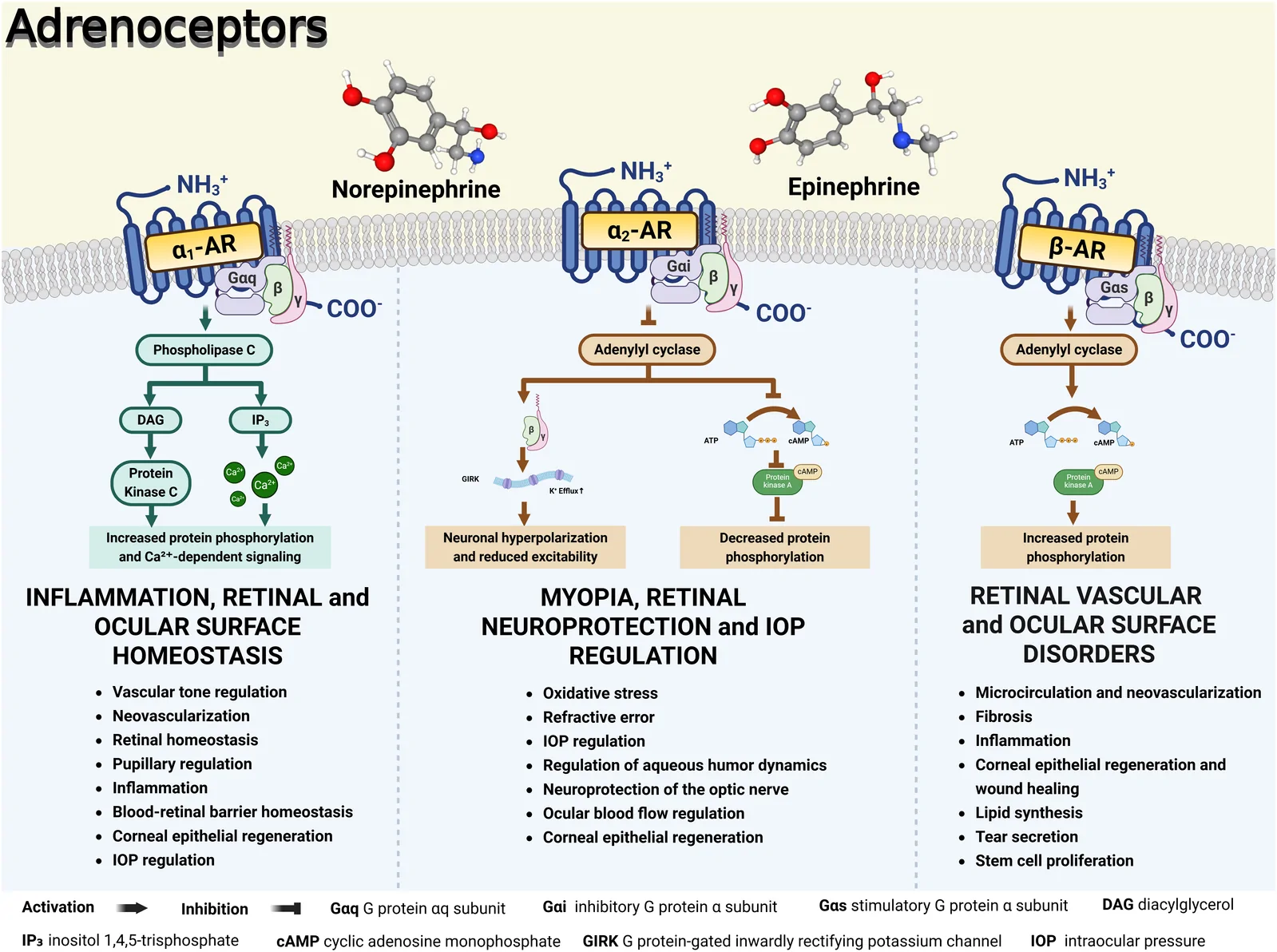
The physiological processes and molecular mechanisms of eye diseases affected by adrenoceptors.

### 2.1 α-AR affects immune inflammation, myopia, and retinopathy

α_2_A-AR represents a promising target for myopia modulation. In form-deprivation myopia (FDM) chicks, intravitreal administration of α_2_-AR agonists Brimonidine, Clonidine, or Guanfacine (200 nM) directly inhibited abnormal axial elongation and refractive error. Conversely, antagonist Yohimbine attenuated deprivation-induced myopia reversal (Carr et al., 2019). Separately, the α_2_-AR agonist Marsanidine (an Imidazoline derivative) induces mydriasis in rats in 1 minute via selective α_2_D-AR activation (rat ortholog of human α_2_A-AR). This effect is blocked by α_2_D-AR antagonist RX821002 (Raczak-Gutknecht et al., 2017), suggesting potential for designing iris-paralyzing myopia therapeutics.

α-ARs significantly contribute to retinal pathology. In experimental diabetic retinopathy, the α_2_-AR agonist brimonidine attenuates retinal ganglion cell (RGC) apoptosis, glial activation, and retinal stress. These effects are associated with restored AKT phosphorylation and BCL-2 expression, inhibition of the p38 MAPK/p53/BAX pro-apoptotic cascade, and reduced GFAP upregulation (Jung et al., 2024), supporting further evaluation of topical brimonidine as a neuroprotective strategy. Conversely, in a high-glucose ARPE-19 cell model, α_1_D-AR antagonist BMY 7378 ameliorates high-glucose-induced mitochondrial impairment and blood-retinal barrier (BRB) dysfunction (Giuffrida et al., 2025), indicating α_1_D-AR blockade protects retinal pigment epithelium (RPE) in early diabetic retinopathy. In a mouse model of autoimmune retinal inflammation, bright light preserves BRB integrity by downregulating α_1_A-AR expression, thereby reducing norepinephrine signaling and immune cell infiltration (Stofkova et al., 2019), positioning local adrenergic modulation as a therapeutic strategy for autoimmune ocular disorders. Sympathetic signaling via α_1_A-AR directly activates eosinophils, with antagonist Tamsulosin diminishing eosinophil responses and inflammation (Liu et al., 2020), suggesting α_1_A-AR targeting for allergic conjunctivitis treatment; corneal γδ T-cells in inflammatory states also express this receptor (Xiao et al., 2019). In a rat experimental retinal detachment model, both α_1_-AR antagonist Doxazosin and α_2_-AR agonist Guanabenz reduce photoreceptor apoptosis and preserve retinal structure/function by inhibiting NADPH oxidase-mediated ROS overproduction, decreasing IL-1β/CCL2, and suppressing gliosis (Li et al., 2019), revealing therapeutic targets for vision preservation. Finally, ex *vivo* studies of rat retinal arterioles showed that norepinephrine induces vasoconstriction via concurrent α_1_A-AR and α_1_D-AR stimulation (Mori et al., 2011), indicating both regulate retinal vascular tone similarly to peripheral circulation.

In summary, α-adrenoceptors critically regulate ocular disease pathogenesis and progression; their dysregulation disrupts intraocular physiological homeostasis, thereby driving pathological processes in multiple ocular diseases.

### 2.2 β-AR is more focused on hypoxia and neovascular proliferative diseases

The retina exhibits high oxygen sensitivity, controlling cellular and molecular processes during physiological and pathological vascular development. β_3_-AR represents the most oxygen-responsive adrenoceptor subtype, functioning as a molecular hub linking anoxia sensing to pathological VEGF generation and vascular proliferation. It represents a preclinical candidate therapeutic target for hypoxic retinal neovascularization (e.g., retinopathy of prematurity). Retinal hypoxia directly upregulates β_3_-AR expression by stabilizing hypoxia-inducible factor-1 (HIF-1), establishing it as a key HIF-1 target gene and core molecule connecting oxygen sensing to neovascularization (Amato et al., 2022; Cammalleri et al., 2023). *In vitro* studies using human retinal endothelial cells (hRECs) and the Müller cell line MIO-M1 showed that β_3_-AR activation enhances VEGF production via NO signaling, supporting a mechanistic role for β_3_-AR signaling in HIF-1-driven VEGF upregulation (Lucchesi et al., 2025). In a mouse oxygen-induced retinopathy (OIR) model, hypoxia upregulates β_3_-AR during pathological neovascularization, while treatment with the β_3_-AR agonist BRL37344 showed beneficial effects on angiogenic signaling, BRB-associated markers, astrocyte density, retinal perfusion, and ganglion cell survival in the preclinical model These preclinical findings suggest that β_3_-AR modulation may involve a negative-feedback mechanism associated with the HIF-1/β_3_-AR axis. These apparently divergent findings suggest that β_3_-AR signaling may be highly context dependent. The pro-angiogenic effect observed in isolated retinal endothelial and Müller cells may not directly reproduce the integrated response of the hypoxic retina *in vivo*, where ligand exposure, disease stage, cellular interactions, and feedback regulation of the HIF-1/β_3_-AR axis may alter the net biological outcome. Differences in agonist concentration and treatment timing may also contribute to the opposing observations. The precise conditions determining pro- versus anti-angiogenic β_3_-AR signaling therefore remain to be clarified. β_2_-AR signaling drives choroidal neovascularization (CNV) by upregulating VEGF and IL-6 expression. Intravitreal β_2_-AR antagonists reduce CNV area and attenuate neovascularization by inhibiting VEGF/IL-6 pathways (Lavine et al., 2017), suggesting therapeutic potential for CNV and retinal neovascularization. In a genetic OIR model, β_1_/β_2_-AR deletion in mice prevents hyperoxia-induced vascular occlusion and pathological neovascularization (Dal Monte et al., 2014), supporting a role for β-AR signaling in hypoxia-associated retinal angiogenesis. Collectively, these studies support β-ARs as candidate therapeutic targets for proliferative retinopathy and highlight the anti-angiogenic value of β-blockers.

Preclinical studies and early clinical investigations have evaluated β-blockers, including propranolol and timolol, for their potential anti-angiogenic effects in ocular vascular disorders, including severe infantile hemangiomas (Léauté-Labrèze et al., 2008), retinopathy of prematurity (Filippi et al., 2013; Filippi et al., 2017), and retinal vein occlusion (Obeid et al., 2021). Topical propranolol partially ameliorates hypoxic retinal vascular injury in experimental models by reducing intraocular VEGF and increasing soluble VEGFR-1, with astrocyte protection contributing to its mechanism (Qadri et al., 2021). Hypoxia drives angiogenesis via β_2_-AR-mediated HIF-1α stabilization and VEGF upregulation, and propranolol attenuates neovascularization in experimental models by interfering with this signaling cascade (Ristori et al., 2011). Separately, early clinical studies in preterm infants with ROP have evaluated propranolol as a potential adjunctive therapeutic approach (Cammalleri et al., 2024; Makhoul et al., 2013). These findings suggest that β-AR blockade may attenuate retinal angiogenesis and BRB dysfunction, while topical delivery has been explored as a strategy to reduce systemic exposure. Propranolol also reduced choroidal neovascularization (CNV) in preclinical models (J. A. Lavine et al., 2013), with subsequent studies suggesting a sex-dependent, macrophage-associated mechanism in female mice (Droho et al., 2019).

Beyond these roles, β-ARs participate in ocular surface and degenerative pathologies, though these areas remain understudied. β_2_-AR signaling resembles α{sb}1{/sb}A-AR in being essential for corneal epithelial regeneration; loss of both causes corneal epithelial thinning and reduced cell volume. β_2_-AR blockers like Levobunolol inhibit β_2_-AR, downregulating EGFR and ERK1/2 phosphorylation via Ki67 expression modulation. They reduce limbal stem cell markers CK3, CK14, and CK19 expression while inhibiting migration and proliferation, ultimately impeding corneal epithelial wound healing (Yuan et al., 2021) and diminishing regeneration-related signaling. β-AR agonists attenuate hyperglycemia-induced decreases in RvD1, 5-LOX, 15-LOX-1, 15-LOX-2, FPR2, and GPR32 in diabetic retinopathy, reversing inflammation (Shi et al., 2017). β-AR imbalance further disrupts ocular surface homeostasis through tear secretion restoration, lipid metabolism, and immune regulation. Studies using human meibomian gland epithelial cells (HMGECs) show that ADRB2/PKA signaling activation enhances meibomian gland lipid synthesis (Jun et al., 2022), supporting a mechanistic role for ADRB2/PKA signaling and its potential as a candidate therapeutic pathway in β-AR blocker-induced meibomian gland dysfunction. Although α_1_-AR overactivation mediates lacrimal mitochondrial dysfunction and inflammation, impairing the NE-α_1_A-AR-mitochondrial bioenergetic axis and reducing tear secretion (Zhang et al., 2023), the NE-β_2_-AR axis directly activates corneal NF-κB pathways. Sympathetic activation drives dry eye via NE-β_2_-AR signaling (Qu et al., 2025); these findings illustrate the complexity of tear secretion regulation and adrenoceptor diversity, warranting further investigation into α_1_-AR and β_2_-AR roles in regulatory axes.

In glaucoma pathogenesis, β_2_-AR autoantibody (β_2_-agAAb) is detected in serum and aqueous humor of >90% of open-angle glaucoma (OAG) patients. Its activity correlates significantly with retinal microcirculation abnormalities, implicating antibody-mediated vasoregulation interference in disease progression (Hohberger et al., 2021a, Hohberger et al., 2021b), supporting β_2_-agAAb as a candidate glaucoma-associated biomarker. In contrast, β_2_-AR signaling itself may represent a candidate therapeutic pathway for glaucoma intervention. Therapeutically, α_2_A-AR agonists (e.g., Brimonidine) suppress inflammatory aqueous flare while lowering intraocular pressure (IOP) through Src family kinase/MMP-mediated EGFR transactivation and enhanced ERK/AKT phosphorylation (Harun-Or-Rashid and Hallböök, 2019; Nakamura-Shibasaki et al., 2016). Novel β-AR/carbonic anhydrase dual-target inhibitors demonstrate superior IOP reduction versus single agents or conventional combinations (Nocentini et al., 2018). Future non-surgical glaucoma therapies should pursue high efficacy, low side effects, and improved adherence, addressing monotherapy limitations in IOP control.

## 3 Dopamine receptors: pleiotropic regulators in multiple ocular diseases

Dopamine receptors (DRs), which are important ARh-GPCRs primarily activated by the endogenous catecholamine dopamine (DA), comprise two major classes: D1-like (D_1_R, D_5_R) and D2-like (D_2_R, D_3_R, and D_4_R), totaling five subtypes. D1-like receptors predominantly couple to Gαs, while D2-like receptors mainly couple to Gαi/o (Beaulieu and Gainetdinov, 2011). In ocular tissues, DRs localize predominantly to retinal neurons and multiple cell types (Zhou et al., 2017). These receptors participate in ocular diseases, including myopia, glaucoma, and retinal degeneration, by mediating retinal neural circuits, visual signal modulation, intraocular pressure adjustment, and ocular growth. The roles of dopamine receptors in regulating biological functions and signaling pathways underlying eye diseases were summarized and presented in **Figure 3**. D_1_R acts as a core hub to regulate neural excitation and photoreadaptation while inhibiting myopia; D_2_R controls the circadian rhythm and its antagonists can repair degenerative lesions. The two have an antagonistic effect in glaucoma, and D_4_R shows potential in preventing myopia, jointly forming a multi-target treatment network.

**Figure 3:**
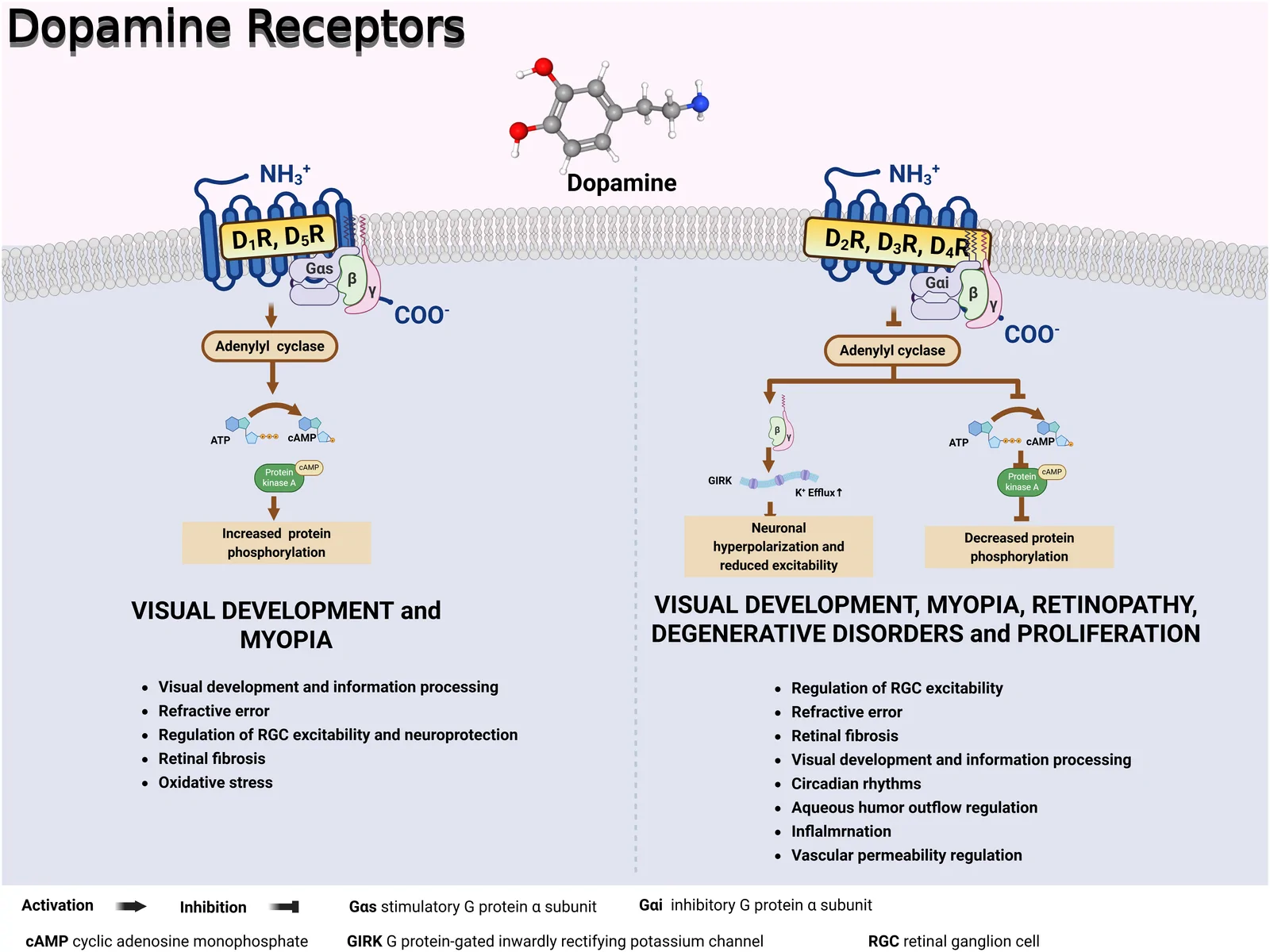
The roles of dopamine receptors in regulating biological functions and signaling pathways underlying eye diseases.

### 3.1 D_1_R regulates retinal adaptation, neural signaling, and ocular growth

D_1_R, the most studied dopamine receptor subtype, mediates light visual signaling, RGC modulation, and myopia. D_1_R mimics light adaptation by modulating inhibitory transmission from presynaptic amacrine to bipolar cells, reducing inhibitory photo-response amplitude and spontaneous inhibitory current frequency—a key retinal mechanism for luminance adaptation and visual information optimization (Flood et al., 2018; Mazade et al., 2019). Studies show D_1_R activation inhibits inwardly rectifying potassium currents (Kir) via the cAMP/PKA pathway, enhancing RGC excitability independent of presynaptic input (Cui et al., 2017). Concurrently, D_1_R activation specifically suppresses Gb/4-AP-sensitive-resistant outward potassium currents through PKA/CaMKII pathways, modulating RGC excitability independently of PKC or MAPK/ERK pathways (Li et al., 2016). Although D_2_R activation also enhances RGC excitability, its agonists act via dual mechanisms: inhibiting Gb/4-AP-sensitive outward potassium currents and enhancing Nav1.6 voltage-gated sodium currents, dependent on cAMP/PKA, CaMKII, and MAPK/ERK signaling but not PLC/PKC (Yin et al., 2020) Bright light-abundant D_1_R synergizes with photoreceptor-enriched D_4_R to reduce RGC input for wide dynamic gain control (Flood and Eggers, 2021).

Dopamine receptors exhibit region-specific myopia regulation. Single-cell sequencing confirmed that anterior scleral DRD1 inhibits remodeling while posterior DRD2 antagonizes this effect; DRD4 silencing enhances dopamine inhibition (Guan et al., 2025), providing new insights into scleral remodeling regulation. Intraperitoneal D_1_R agonist SKF38393 attenuated form-deprivation myopia (FDM) in mice, whereas antagonist SCH39166 promoted myopia (Shu et al., 2023). However, DRD1 knockout (Drd1-KO) and retinal conditional knockout (Drd1-CKO) mice still developed FDM with refractive status matching wild-type, indicating D_1_R is a key myopia-inhibiting target but not essential for refractive development. Peribulbar SKF38393 injections in guinea pigs significantly suppressed myopia, showing cross-species D_1_R agonist protection (Jiang et al., 2014; Zhang et al., 2018). ON/OFF signaling imbalance in cone bipolar cells and dopamine receptor downregulation in highly myopic retinas lacking long-synaptic cells further confirm abnormal light processing during myopia progression (Yao et al., 2023). Conversely, intense light (2500–5000 lux) activated D_1_R in ON-bipolar cells, significantly inhibiting FDM in mice (Chen et al., 2017). Collectively, these findings underscore intrinsic retinal dopaminergic mechanisms in ocular growth regulation, prompting further exploration of dopaminergic orthopia control pathways.

### 3.2 In addition to regulating vision, D_2_R is also involved in processes such as inflammation and fibrosis

In the vertebrate eye, physiological D_2_R regulates both eye development/visual function and retinal circadian rhythms. Zebrafish studies show D_2_R antagonists impair eye development and visual function, attenuated by the agonist Quinpirole (Syambani Ulhaq, 2020). In mammals, D_2_R regulates RPE circadian rhythms by specifically inducing period1/period2 clock genes (Baba et al., 2017), suggesting therapeutic potential for rhythm-related eye diseases. Retinal dopaminergic neuron pathway dysfunction may cause visual abnormalities, particularly given dopamine’s role in light-circadian regulation.

D_2_R affects myopia more complexly than D_1_R, with overall pro-myopic effects but dose- and model-dependent variations. Form-deprivation myopia (FDM) attenuated significantly in Drd2-KO mice and Sulpiride-treated wild-types (Huang et al., 2022; Wei et al., 2023a); D_2_R antagonism also alleviated FDM in guinea pigs by modulating RPE/choroidal proline/arginine metabolism pathways (Wei et al., 2023b). Low-dose Quinpirole inhibits myopia while high doses promote progression, indicating complex dose-dependent regulation (Huang et al., 2020). Intriguingly, intravitreal Quinpirole (D2-like agonist) and Spiperone (antagonist) both inhibited myopia in tree shrews, as did D_4_R agonist PD168077 (Ward et al., 2017), suggesting functional overlap between D_2_R and D_4_R. These seemingly contradictory pharmacological effects may reflect differences in ligand concentration, receptor-subtype selectivity, and experimental species or myopia models. In particular, Quinpirole and other D2-like ligands are not absolutely restricted to a single dopamine receptor subtype, and dose-dependent recruitment of different receptor populations or downstream pathways may produce distinct effects on ocular growth. Therefore, the available findings support context-dependent dopaminergic regulation rather than a simple unidirectional role for D2R in myopia.

D_2_R further serves as a critical pathological mediator and therapeutic target in retinopathy by regulating inflammation, excitatory neurotransmission, light processing, and fibrosis. Early diabetic retinopathy features dopamine-decreased D_1_R/D_4_R inactivation, loss of retinal ERG response, and receptor-mediated neurological dysfunction (MacIsaac et al., 2024), indicating altered dopamine signaling as a key mechanism. In chronic high-IOP glaucoma, D_2_R activation enhances RGC hyperexcitability damage, while D_1_R activation or D_2_R antagonism attenuates apoptosis (Yin et al., 2023), revealing D_1_R-D_2_R antagonism where D_1_R is neuroprotective and D_2_R promotes injury, making D_2_R inhibition or D_1_R activation viable glaucoma treatments. Degenerative retina dopaminergic alterations worsen RGC photo-responses; Sulpiride (D_2_R antagonist) corrects photosensitivity/latency abnormalities in retinitis pigmentosa, reducing ON responses while inducing short-latency OFF responses to repair signaling codes (Jensen, 2015). For retinal fibrosis, D_2_R inhibition or D_5_R activation suppresses TGF-β-triggered pro-fibrotic gene expression, migration, proliferation, and fibronectin deposition, offering new targets for proliferative vitreoretinopathy (PVR) (Gao et al., 2022).

### 3.3 D_3_R, D_4_R, and D_5_R are still understudied in current eye disease research

The research on D_3_R, D_4_R and D_5_R is still limited and mainly focuses on myopia. The mechanism of action remains unclear. D_4_R agonists have shown similar anti-myopia effects in tree shrews (Ward et al., 2017), but direct mechanism studies on D_4_R and D_5_R are still lacking. Current drug intervention measures (such as apomorphine, quipiro) target the dopamine receptor family rather than specific subtypes, which complicates signal attribution and makes it impossible to draw clear conclusions about dopamine signal transduction in eye growth driven by vision (Zhou et al., 2017).

The key breakthrough in recent years lies in single-cell sequencing, which revealed that D_4_R is highly expressed in posterior scleral fibroblasts of the human sclera. This is the first to provide a histological basis for D_4_R’s direct regulation of scleral remodeling and its involvement in eye axis elongation (Guan et al., 2025). Moreover, considering the known role of D_4_R in regulating the circadian rhythm of the retina and light adaptation (Flood and Eggers, 2021), its potential role in connecting environmental light and eye growth signals deserves in-depth exploration. However, research on D_3_R is nearly non-existent, and D_5_R is difficult to distinguish due to its high homology with D_1_R. Future research urgently needs to utilize gene knockout models and highly selective tools based on structural design to elucidate the precise roles of these subtypes in the retinal-scleral signaling axis.

## 4 Muscarinic cholinergic receptors at the forefront of research on receptors for myopia-predominant ocular pathologies

Muscarinic receptors (MRs), belonging to the amine-responsive GPCR family, bind endogenous acetylcholine (ACh). Five subtypes (M_1_R-M_5_R) are identified: M_1_R, M_3_R, and M_5_R primarily activate Gαq/11, while M_2_R and M_4_R mainly couple to Gαi/o. MRs are distributed predominantly in the pupillary sphincter, ciliary muscle, and retinal layers, regulating pupillary reflexes, lens accommodation, and retinal signaling (Ruan et al., 2021). They also participate in ocular disease pathogenesis, including glaucoma, myopia, and retinal degenerative diseases. Acetylcholine receptors involvement in the functional modulation and intracellular mechanisms of ocular diseases were summarized and presented in **Figure 4**.

**Figure 4:**
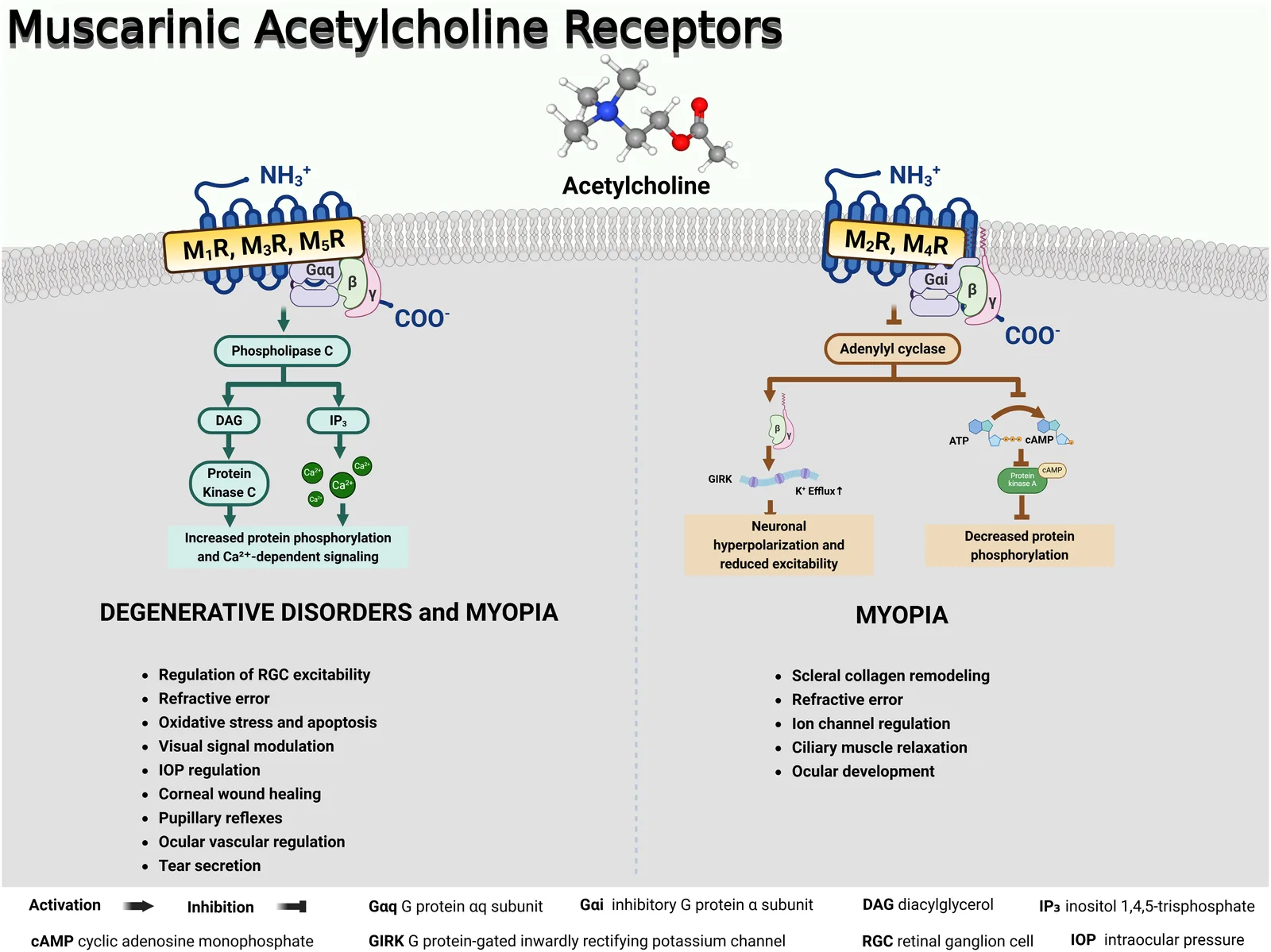
Acetylcholine receptors involvement in the functional modulation and intracellular mechanisms of ocular diseases.

### 4.1 M_1_R is focused on glaucoma and refractive errors

M_1_R mediates retinal neuroprotection by regulating oxidative stress and apoptosis pathways, promoting retinal neuron survival, and represents a potential glaucoma therapy target with age-dependent effects. M_1_R activation promotes retinal neuron survival; however, young M1R-deficient mice show no differences in RGC layer (RGCL) cell counts or optic nerve axon numbers versus wild-type mice. Only M4R mRNA compensatory upregulation occurs (Laspas et al., 2015), indicating M1R is non-essential for retinal neuron survival *in vivo*. In contrast, aged M_1_R-deficient mice exhibit significant RGC loss via oxidative stress dysregulation, characterized by elevated pro-oxidant NOX2 expression, reduced antioxidant enzymes (SOD1, GPx1, HO-1), and consequent ROS accumulation (Laspas et al., 2019), supporting M_1_R activation’s therapeutic relevance for RGC survival. This discrepancy accentuates M_1_R’s age-dependent neuroprotective role. Huperzine A (HupA) indirectly activates M_1_R via acetylcholinesterase inhibition and elevated acetylcholine, activating AKT signaling while inhibiting MAPK to attenuate oxidative stress (increased GSH/SOD, decreased MDA) and apoptosis (reduced Bax/Bcl-2 ratio and caspase-3 activity), while exerting IOP-lowering effects through M_3_R activation, constituting a dual glaucoma protective mechanism (Yu et al., 2021). Similarly, Pilocarpine’s neuroprotection is IOP-independent and attenuates RGC damage *in vivo* and *in vitro* by downregulating caspase-3 via M_1_R activation and upregulating cholinergic marker ChAT (Tan et al., 2014). Muscarinic receptor activation directly stimulates vulnerable ipRGCs in glaucoma, triggering intense discharges (Sodhi and Hartwick, 2016), revealing mAChR-mediated photoreceptor stimulation of ipRGCs as a novel strategy for targeting ipRGCs to intervene in glaucomatous RGC damage.

Muscarinic receptors also mediate myopia development by modulating downstream signaling, and their antagonists represent an effective intervention strategy. Muscarinic receptor signaling drives myopic growth through transglutaminase TGM-2 activation. Specifically, M_1_R, M_4_R, and M_5_R directly contribute to ocular axial elongation via TGM-2 upregulation (Barathi et al., 2013), indicating that TGM-2 and muscarinic receptors play central roles in myopia pathogenesis. Blocking these factors may interfere with myopia progression, constituting at least part of the anti-muscarinic drugs’ therapeutic mechanism. Both highly selective M_1_R antagonist MT7 and M_4_R antagonist MT3 significantly inhibited form-deprivation myopia in tree shrews, with M_1_R antagonism also deterring lens-induced myopia (Arumugam and McBrien, 2012). Green flickering light-induced myopia in guinea pigs was accompanied by significant M1R mRNA downregulation (Tao et al., 2018), further suggesting that M1R signaling is associated with myopia development. However, given that muscarinic antagonism can also inhibit experimental myopia, the precise functional role and directionality of M1R signaling in ocular growth remain unresolved.

### 4.2 M_2_R, M_3_R, and M_4_R are focused on ionic homeostasis and genetic regulation of myopia

Regarding myopia, M_2_R deletion or blockade inhibits myopia development by increasing scleral collagen-I and decreasing collagen-V expression, revealing M_2_R regulates scleral collagen in myopia pathogenesis (Barathi et al., 2023). This receptor also modulates myopia progression by upregulating retinal Kir3.4 channels and reducing K^+^ concentration (Zhou et al., 2025). M_3_R copy number variation (CNV) significantly correlates with high myopia (Lin et al., 2012).

Notably, atropine, although classically characterized as a nonselective muscarinic antagonist, may also engage non-muscarinic mechanisms at concentrations relevant to experimental myopia inhibition. *In vitro* findings indicate that α_2_A-AR inhibition requires relatively high atropine concentrations (≥0.1 mM), which overlap with the effective dose range reported in chick models (Carr et al., 2018). These observations raise the possibility that atropine-induced inhibition of ocular growth may not be mediated exclusively through muscarinic receptor blockade. The apparently different pharmacological interpretations may reflect concentration-dependent off-target effects, incomplete receptor-subtype selectivity, species-related differences in receptor pharmacology, and variation in ocular tissue exposure. Therefore, the relative contributions of muscarinic and non-muscarinic targets to atropine-mediated myopia control remain unresolved.

In ocular surface diseases, M_3_R⁻/⁻ mice exhibit impaired tear production, progressing to dry eye disease (DED) in aging with corneal pathology, goblet cell loss, and oxidative inflammation (Musayeva et al., 2021), indicating M_3_R’s critical role in tear secretion and DED manifestations. For vascular regulation, M_3_R mediates bidirectional ophthalmic artery responses: endothelium-dependent vasodilation (ACh-concentration-dependent with intact endothelium) and endothelium-independent vasoconstriction. This dual regulation is abolished in M_3_R⁻/⁻ mice (Gericke et al., 2014).

Notably, classical myopia treatment atropine may primarily block α_2_A-AR rather than the traditionally targeted M_4_R, challenging established targeting paradigms. However, M_4_R/M_5_R research is limited by selective tool compound scarcity, low ocular expression, and skewed research focus. Future work needs to develop specific probes and gene-edited models to resolve M_4_R’s role in neural compensation and M_5_R in ocular development, design tissue-targeted delivery systems based on subtype spatial distribution, and explore multi-subtype synergistic strategies to advance cholinergic networks for ophthalmic precision medicine.

## 5 Histamine receptors as “triple guards” for IOP homeostasis, vascular homeostasis, and immune homeostasis

Histamine, a biogenic amine secreted from mast cells and basophils, acts through histamine receptors (HRs). Four receptor subtypes exist: H_1_R (predominantly Gαq/11-coupled), H_2_R (mainly Gαs-coupled), H_3_R, and H_4_R (primarily Gαi/o-coupled, sharing high homology). These receptors contribute to multiple ocular pathologies by regulating aqueous production, retinal neurotransmission, vascular tone, light signaling, and local immune surveillance (Sgambellone et al., 2021).

The intraocular histaminergic system primarily regulates IOP homeostasis and its circadian fluctuations (Lanzi et al., 2019). H_1_R and H_3_R bidirectionally modulate IOP homeostasis and retinal neuroprotection, supporting their potential as candidate therapeutic targets for glaucoma. H_1_R activation promotes RGC death via endoplasmic reticulum calcium release and pro-apoptotic CHOP expression; its antagonist Maprotiline blocks this pathway, inhibits endoplasmic reticulum stress, restores ER homeostasis, and protects RGCs/visual function in injury models (Chen et al., 2022). Conversely, H_3_R antagonist Ciproxifan combined with NO donors ST-1989/ST-2130 lowers IOP through dual mechanisms: antagonizing H_3_R to promote endogenous histamine release while activating the H_1_R-PLC-Ca²-eNOS pathway to enhance NO production and improve aqueous outflow (Sgambellone et al., 2024). These apparently divergent effects of H1R signaling may reflect differences in cellular and tissue context rather than a uniformly protective or detrimental role of H1R. Whereas H1R activation in RGCs has been associated with ER-stress-related neurotoxicity, H1R-dependent PLC-Ca²^+^-eNOS signaling in the context of H3R antagonism and NO donation is linked primarily to IOP regulation and aqueous outflow. Thus, the biological consequences of histaminergic signaling may depend on the responding cell type, downstream pathway engaged, and experimental context. Such dual-action compounds represent novel strategies against RGC loss in glaucoma and retinal diseases. Chronic H_3_R antagonism also improves ocular arterial function, attenuates oxidative stress, reduces RGC death, and synergistically protects the optic nerve by modulating IOP circadian rhythm (nocturnal dip/morning peak) (Lanzi et al., 2019). Thus, H_1_R-targeted neuroprotection and H_3_R-targeted IOP modulation form complementary strategies, expanding precise glaucoma therapeutics.

Available evidence suggests that H_4_R may contribute to vascular permeability and pro-angiogenic signaling in diabetic retinopathy (DR).H_4_R activation mediates macrophage chemotaxis and vascular leakage in diabetic patients with elevated serum/vitreous histamine, increasing vascular permeability (Kwon et al., 2024). Hyperglycemia induces H_4_R overexpression in ARPE-19 cells, upregulating VEGF/IL-6 while inhibiting PEDF via the p38 MAPK pathway, promoting abnormal vascular proliferation. H_4_R knockdown attenuates this imbalance (Lee et al., 2020), supporting a mechanistic role for H4R signaling and identifying H4R as a preclinical candidate target that warrants further investigation in DR.H_1_R and H_4_R critically regulate orbital immune inflammation. H_1_R mediates mast cell-driven inflammation: orbital infiltrating mast cells release histamine, selectively activating fibroblast H_1_R and upregulating IL-6/IL-8/CCL2 via NF-κB pathway. The H_1_R antagonist Loratadine concentration-dependently inhibits >90% cytokine production, and targeted H_1_R blockade suppresses Graves’ ophthalmopathy inflammation (Virakul et al., 2016). H_4_R activation enhances macrophage chemotaxis and phagocytosis, blocked by H_4_R antagonist JNJ7777120, which attenuates inflammatory infiltration/endothelial permeability and prevents macrophage infiltration/retinal leakage (Czerner et al., 2014). Thus, H_1_R/H_4_R signaling modulates mast cell/macrophage-mediated inflammatory infiltration and pathogen clearance, underlying pathologies like allergic conjunctivitis and autoimmune ophthalmopathy, and providing a rationale for anti-inflammatory and anti-immune disorder therapies targeting histamine receptors.

## 6 Serotonin receptor regulatory network involves facets from neurons to tumor cells

The diverse biological effects of serotonin (5-HT) are mediated through serotonin receptors (5-HTRs). This receptor family comprises seven classes with over 14 subtypes, predominantly GPCRs except 5-HT_3_R. Specifically, 5-HT_1_R and 5-HT_5_R couple to Gαi/o, 5-HT_2_R to Gαq/11, and 5-HT_4_R, 5-HT_6_R, and 5-HT_7_R to Gαs (Costagliola et al., 2008). In ocular tissues, 5-HTRs localize primarily to the ocular surface, aqueous pathway, and retina. They participate in neuronal visual processing, vascular tone regulation, IOP modulation, and tumor migration, and are implicated in degenerative glaucoma, diabetic retinopathy, and uveal melanoma.

5-HT_1_AR agonists mediate neuroprotection by modulating the excitation-inhibition balance. In glaucoma models, elevated IOP downregulates retinal 5-HT_1_AR. Its agonist 8-OH-DPAT enhances RGC survival via receptor activation, antagonizing excitotoxicity through cAMP-PKA inhibition, presynaptic GABA release facilitation, and inhibitory synaptic current (sIPSCs/mIPSCs) potentiation. This neuroprotection is fully blocked by antagonist WAY-100635 or GABAA blocker SR95531 (Zhou et al., 2019a, Zhou et al., 2019b), supporting 5-HT_1_AR as a candidate neuroprotective therapeutic target in preclinical glaucoma models.

Uveal melanoma (UM), the most common primary intraocular malignancy in adults, exhibits hepatic metastasis closely linked to HTR2B aberrant activation (Le-Bel et al., 2019). HTR2B overexpression is dynamically regulated at multiple levels. Transcriptionally, IL-4/IL-6 enhances HTR2B transcription via JAK/STAT-mediated STAT1 promoter binding (Benhassine et al., 2022), while activator NFI and repressor RUNX1 competitively bind antagonist elements, causing expression imbalance (Benhassine and Guérin, 2018), accumulating HTR2B mRNA. Post-translationally, ubiquitinated HTR2B evades ubiquitin-proteasome system (UPS) degradation in metastatic UM cells, with proteostasis imbalance correlating significantly with metastatic potential (Le-Bel et al., 2019). Functionally, HTR2B overactivation drives WNT/β-catenin, FAK, and JAK-STAT pathways via phosphorylation cascades, promoting UM cell proliferation, clonogenicity, migration, and invasion (Weidmann et al., 2018). Preclinical studies indicate that selective HTR2B antagonists can suppress HTR2B-associated signaling networks and tumor-related phenotypes, supporting HTR2B as a candidate therapeutic target for UM. Multi-target approaches involving the STAT1-HTR2B axis, NFI/RUNX1 balance, and UPS regulation may warrant further investigation as potential therapeutic strategies.

Serotonin receptors regulate visual development and plasticity via spatiotemporal-specific mechanisms. Juveniles exhibit 5-HT_2_BR-mediated microglial synaptic pruning with 2.1-fold increased convergence rate and precise retino-thalamic projection spatial coordination; its absence causes disorganized projections and inflammatory marker abnormalities (Kolodziejczak et al., 2015). Concurrently, retinal 5-HT_2_CRs inhibit ganglion cell synaptic growth/RGC activity, forming localized serotonin loops from amacrine cells. 5-HT_2_CR KO mice show an abnormally elevated pattern of electroretinogram (ERG) amplitudes (Trakhtenberg et al., 2017). In adulthood, 5-HT_2_AR and 5-HT_3_AR mediate cross-modal cortical remodeling post-visual deprivation (Lombaert et al., 2018), while serotonin transporter SERT modulates visual processing by selectively inhibiting ON-OFF RGC excitatory transmission and reducing spontaneous excitatory postsynaptic current (sEPSC) frequency - effects interfered by SSRIs (Di Berardino et al., 2025). Notably, retinal serotonin networks demonstrate bidirectional pathology: 5-HT_2_Rs exacerbate inner retinal layer damage, whereas 5-HT_1_AR protects photoreceptors (Masson, 2019).

Serotonin receptor subtypes exert disease-specific regulatory roles in diverse ocular pathologies. The 5-HT_2_AR antagonist Ritanserin restores corneal transparency in aniridia by activating PAX6 through MEK/ERK pathway inhibition, repairing impaired limbal stem cell migration (Oved et al., 2021), offering potential blindness treatment via corneal clarity restoration. Serotonin receptors complement refractive error pathology, evidenced by retinal 5-HT_2_AR upregulation in flickering light-induced myopia models correlating with myopia severity (Li et al., 2016). In optic nerve repair, 5-HT_1_R signaling promotes early axon regeneration in zebrafish, whereas pharmacological inhibition of 5-HT₁R impairs regenerative axon growth (Saied-Santiago et al., 2025), providing both a mammalian nerve repair targeting strategy and mechanistic evidence for serotonin-dependent neuromodulation directing *in vivo* optic nerve regeneration.

## 7 Melatonin receptors primarily complement temporal rhythm disorder regulation in ophthalmic disease

Melatonin (MT), an important Indoleamine hormone primarily synthesized by the pineal gland and retina, mainly regulates circadian rhythms. Its biological effects are mediated through high-affinity receptors MT_1_R and MT_2_R. These receptors primarily couple to Gαi/o, inhibiting adenylyl cyclase, reducing intracellular cAMP, and suppressing PKA. They also activate PLC, increasing IP_3_/DAG, mobilizing intracellular Ca²^+^, and activating PKC. Additionally, MT_2_R regulates cGMP levels. Melatonin receptors are widely expressed in the retina and cornea, modulating circadian rhythms, antioxidant pathways, anti-inflammatory responses, and cell survival mechanisms (Alarma-Estrany and Pintor, 2007). They centrally protect against uveitis, photoreceptor degeneration, glaucoma, corneal injury, and ocular tumors.

Melatonin receptors (MTRs) disease-associated expression alterations in uveal melanoma. MTNR1A and MTNR1B demonstrate low expression in uveal melanoma (UM) primary tissues, yet MTNR1A significantly elevates in deceased patients (Hagström et al., 2024), supporting further evaluation of MTNR1A as a candidate prognostic biomarker. Conversely, MTR agonists Agomelatine and Ramelteon attenuate endotoxic uveitis (EIU) by reducing inflammatory infiltration and iris vasodilation through TNF-α/VEGF/apoptotic factor inhibition while increasing GSH and SOD and reducing MDA (Yuksel et al., 2023), indicating therapeutic potential for uveitis.

Glaucoma progression involves MT_1_R and MT_2_R attenuation, particularly MT_2_R (Martínez-Águila et al., 2020). Preclinical pharmacological studies suggest therapeutic potential for melatonin receptor-directed strategies. For example, melatonin reprograms Gαs signaling through α_1_A-AR/MT1R/MT2R heterodimers, lowering IOP by 29% when administered alone and enhancing the IOP-lowering efficacy of the α_1_-adrenergic antagonist perphenazine by 45.8% when used in combination (Alkozi et al., 2020). Both melatonin receptor activation and fatty acid amide hydrolase (FAAH) inhibition show therapeutic potential. Dual-target compounds combining MTR agonists and FAAH inhibitors extend IOP-lowering duration during treatment (Spadoni et al., 2018), showing prolonged ocular hypotensive activity in a rabbit model, supporting further investigation of this dual-target strategy for glaucoma.In photoreceptor diseases, transcriptomic-proteomic analysis reveals MT_1_R mediates aberrant scleral clock gene (RORα/RORβ) activation, driving axial elongation (H. Li et al., 2025), linking scleral collagen remodeling to circadian rhythms. MTNR mutations cause 73% tropical Xenopus rod loss, 33% cone reduction, and centrally-preferential retinal degeneration (Wiechmann et al., 2020), indicating that mutant melatonin receptor disruption impairs rod survival. Conversely, MTNR1A/MTNR1B double-knockout mice exhibit 30% senescent cone loss due to disrupted PI3K-AKT-FOXO1 pathway rhythmicity (Gianesini et al., 2016). Melatonin receptor deficiency also accelerates retinal pigment epithelial damage (Laurent et al., 2017), supporting a protective mechanistic role of melatonin receptor signaling and its potential as a candidate therapeutic pathway in age-related macular degeneration.

MT_2_R activation attenuates blue-light corneal epithelial damage by modulating autophagy-apoptosis. MT_2_R agonist IIK7 (N-butanoyl-2-[2-methoxy-6H-isoindolo (2,1-a) indol-11-yl] ethanamine) reduces fluorescein staining, increases tear volume, and accelerates epithelial healing by balancing BAX-mediated autophagy-apoptosis while reducing ROS/CD4^+^IFN-γ^+^ T-cell infiltration to alleviate oxidative stress (Jin et al., 2022; Yoon et al., 2024). The MT_2_R-BAX pathway regulates blue-light-impaired autophagy-apoptosis and corneal damage, highlighting IIK7’s anti-inflammatory role in post-injury immune response attenuation.

## 8 Trace amine-associated receptors are currently a cognitive gap in ophthalmic disease research

Nine mammalian trace amine-associated receptor (TAAR) subtypes (TAAR1-TAAR9) have been identified, primarily activated by endogenous trace amines (TAs), including Phenylethylamine (PEA), Tyramine (TYR), Tryptamine (TRP), and Octopamine (OA) (Gainetdinov et al., 2018). TAAR1, the most studied subtype, couples to Gαs, Gαi/o, and Gαq/11 proteins, while other subtypes’ functions remain unclear.

Current ocular evidence for TAARs is extremely limited and has been reported primarily in the context of primary open-angle glaucoma (POAG). Non-targeted metabolomic analysis identified altered aqueous humor β-phenylethylamine levels associated with POAG, although a causal role has not been established. Rabbit eye studies showed 0.25% β-phenyl-ethylamine (β-PEA) administration significantly induced pupil dilation via TAAR1 activation without altering IOP (Gowtham et al., 2023).

No consensus has yet been established regarding the pathological roles of TAARs in ocular diseases, largely because the available evidence remains scarce, preliminary, and predominantly pharmacological. Endogenous ligands such as β-PEA occur at low intraocular concentrations and undergo rapid metabolism, necessitating highly sensitive analytical approaches for their detection and further complicating ocular TAAR research. At present, the ocular evidence is largely limited to β-PEA-associated metabolomic observations and pharmacological studies in rabbit eyes, in which β-PEA induced TAAR1-dependent pupillary dilation without altering IOP. Therefore, any proposed neuroprotective or IOP-regulatory role of TAAR1 should currently be regarded as a hypothesis requiring direct experimental validation rather than an established therapeutic effect. Further studies using receptor-selective ligands, genetic models, and clinically relevant ocular systems are needed before the translational significance of TAAR1 can be determined.

## 9 Convergence and crosstalk of ARh-GPCR signaling

Although ARh-GPCRs differ substantially in ligand specificity, tissue distribution, and G-protein coupling, many of them converge on a limited set of intracellular signaling modules, including cAMP/PKA, PLC/Ca²^+^/PKC, PI3K-AKT, MAPK, and RhoA-ROCK pathways. Such signaling convergence is well established in the broader GPCR field (Rosenbaum et al., 2009); however, it should not be assumed that all of these interactions have been directly demonstrated in ocular tissues. At present, direct evidence for receptor–receptor crosstalk among ARh-GPCRs in the eye remains relatively limited, whereas evidence for pathway-level and functional convergence is more extensive.

Several ocular studies nevertheless provide direct or functional evidence of receptor crosstalk. At the receptor level, α_1_A-adrenoceptors have been shown to form functional complexes with MT1 and MT2 melatonin receptors in aqueous-humor-producing cells and human ocular tissues. Formation of these complexes alters G-protein coupling, whereas their disruption in experimental glaucoma is associated with altered Ca²^+^ signaling and IOP regulation (Alkozi et al., 2020). Similarly, D3 dopamine receptors form heteromers with MT1 or MT2 receptors in ciliary body epithelial cells and human ocular samples; heteromerization modifies D3R-Gi coupling and MAPK signaling, and reduced heteromer expression has been associated with ocular hypertension (Reyes-Resina et al., 2020). These studies provide some of the clearest evidence that distinct ARh-GPCR families can interact directly in ocular tissues.

More recent studies support additional forms of pathway-level or functional convergence in ocular systems. In corneal limbal stem/progenitor cells, β_2_-AR blockade reduces EGFR and ERK1/2 phosphorylation and impairs epithelial regeneration, indicating functional coupling between β_2_-adrenergic signaling and the EGFR-ERK pathway during corneal wound healing (Yuan et al., 2021). In retinal circuits, D1-like and D2-like dopamine receptor signaling differentially modulates overlapping components of retinal ganglion-cell receptive fields, illustrating subtype-dependent convergence within the same retinal neural network (Warwick et al., 2023). In experimental ocular hypertension, combined H3R antagonism and nitric oxide release influences both histaminergic and nitrergic mechanisms to regulate IOP, retinal perfusion, and neuroprotection, providing an example of cross-system signaling integration in an ocular disease model (Sgambellone et al., 2024). These examples should be distinguished from direct receptor heteromerization because they primarily demonstrate functional or downstream pathway convergence rather than physical receptor–receptor interaction.

Accordingly, the ocular microenvironment may be conceptualized as a signaling network in which multiple GPCRs can converge on shared intracellular nodes; however, a broader ARh-GPCR “signal ecosystem” should currently be regarded as a working framework rather than an established ocular signaling architecture. Recent reviews of retinal cAMP signaling and ocular melatonin biology similarly emphasize interactions among neurotransmitter systems and shared signaling pathways, while also highlighting the limited direct evidence for receptor-level interactions in the eye (Hou and Pan, 2025; Zhang et al., 2024). Future studies using proximity-based receptor-interaction assays, cell-type-specific genetic models, single-cell and spatial multi-omics, and real-time signaling approaches will be required to distinguish direct receptor crosstalk from parallel convergence on common downstream pathways and to determine whether these interactions can be therapeutically exploited in ocular disease.

## 10 Conclusions and future challenges

ARh-GPCRs, which constitute the largest GPCR subfamily, play central roles in ocular disease initiation and progression. As central regulators in critical ocular pathways, these receptors represent highly promising therapeutic targets. By summarizing the research of the past decade, we have concluded that ARh-GPCRs have established a unified pathophysiological framework in terms of the nervous, vascular, immune, and circadian rhythms of ophthalmic diseases. Moreover, these receptors operate not in isolation but within a hierarchically organized “target ecosystem” within these processes, which reveals novel therapeutic paradigms.

From a systemic perspective, despite exhibiting receptor-specific differences, distinct ARh-GPCR subfamilies nevertheless demonstrate significant functional commonalities. Most receptors share key downstream signaling nodes—such as cAMP/PKA, Ca²^+^-dependent pathways, and MAPK—in neurotransmitter release, vascular permeability, inflammatory responses, and circadian rhythm regulation. This suggests they may amplify disease phenotypes through convergent mechanisms under pathological conditions. Differences in their cellular distribution, signaling coupling preferences, and temporal dynamics, however, determine their functional specialization across distinct disease stages and tissue compartments. This ‘shared pathways-differential regulation’ organizational pattern provides a theoretical foundation for multi-target therapy. By synergistically modulating multiple functionally related yet non-redundant GPCR targets, this approach holds promise for enhancing therapeutic efficacy while mitigating the risk of adverse effects associated with excessive single-target intervention. Systems and network pharmacology studies further indicate that multi-target strategies demonstrate superior stability and long-term benefits compared to single-target therapies in complex chronic diseases, offering significant implications for ophthalmic disorders (Hopkins, 2008).

Despite considerable research advances, fundamental challenges persist across methodological, theoretical, and translational domains, substantially impeding mechanistic insight and clinical application. Key technical limitations include inadequate target specificity and signaling pathway resolution, largely attributable to the poor selectivity of existing pharmacological tools. Many subtype-selective ligands have been validated primarily in overexpression systems, yet highly selective antagonists remain scarce due to the high sequence homology among receptor subtypes. This frequently leads to off-target effects in ocular tissues, where multiple homologous receptors are co-expressed. For example, the dose-dependent bidirectional effects of the D_2_R agonist Quinpirole, alongside evidence that atropine may exert anti-myopic effects via α_2_A-AR rather than muscarinic receptors, underscore the limited reliability of conventional pharmacological approaches. Furthermore, most genetic knockout models are constitutive rather than cell type-specific, constraining functional insights into receptor roles across diverse ocular cell populations—such as retinal neurons, pigment epithelial cells, and vascular endothelial cells—as well as across developmental stages. Current pharmacological tools also lack spatiotemporal precision, hindering the investigation of dynamic GPCR signaling in disease contexts. Additional translational barriers include species differences in receptor expression and function, along with the limited reproducibility of disease models. Furthermore, the key experimental evidence in this field currently derives primarily from non-human animal models such as chickens, mice, and guinea pigs. However, G protein-coupled receptors may exhibit variations across species in terms of their expression profiles, spatial distribution, signal coupling characteristics, and the cross-regulatory patterns within pathway networks. Consequently, conclusions regarding receptor function and pharmacology drawn from animal models may not be directly extrapolated to human ocular physiology and pathology. This uncertainty is particularly pronounced in chronic ocular diseases such as myopia, glaucoma, and retinal degeneration, where human conditions exhibit longer disease progression, more complex genetic and environmental backgrounds, and distinct developmental and compensatory mechanisms compared to animal models, thereby amplifying the challenges of interspecies translation. Finally, research efforts remain fragmented, often focusing on individual receptors within isolated disease models without systematic comparison—such as assessing the contributions of multiple receptor subtypes within a unified model—or integrative analysis of cross-receptor family synergies. These gaps collectively obstruct a holistic understanding of ocular GPCR networks as integrated systems governing physiology and pathology.

To overcome these limitations, first and foremost, there is an urgent need to systematically validate and refine findings from animal studies by integrating multi-omics data from human ocular tissues, humanized models, and organoids derived from induced pluripotent stem cells. This approach will enable a more prudent delineation of the clinical translational potential and applicable boundaries of GPCR-related mechanisms. Secondly, future research should transition from the traditional ‘single receptor-single disease’ paradigm towards a precision pharmacology framework guided by disease phenotypes and temporal dynamics. For instance, in diabetic retinopathy, combined administration of H_4_R antagonists and β_2_-AR blockers may enable multi-stage interventions spanning early vascular leakage to late proliferative phases. In glaucoma, co-targeting MT_1_R/MT_2_R agonists and H_3_R antagonists could synergistically reduce intraocular pressure while protecting RGC. Ultimately, advancing GPCR-targeted ophthalmic therapies will require integrating structural pharmacology with multi-omics technologies to systematically map receptor interaction networks, thereby providing a basis for next-generation GPCR-targeted therapeutic strategies in ocular diseases.
